# A Case Report with Severe Thrombocytopenia Induced by Axitinib

**DOI:** 10.1155/2020/7520783

**Published:** 2020-02-07

**Authors:** Ulkuhan I. Koksal, Janeiro Valle Goffin, Brian Lewis, Oliver A. Sartor, Elizaveta Belyaeva, Francisco Socola, Pedro C. Barata

**Affiliations:** Tulane Cancer Center, School of Medicine, Tulane University, New Orleans, LA, USA

## Abstract

Axitinib is an oral, second-generation tyrosine kinase inhibitor that is selective for vascular endothelial growth factor receptors (VEGFR). This agent is approved as monotherapy or in combination with immune checkpoint inhibitors for the treatment of metastatic renal cell carcinoma. Axitinib is associated with a safety profile very similar to other anti-VEGFR inhibitors but usually with fewer hematologic adverse events, due to the selectivity for VEGF. In this report, we presented a rare case of grade 4 axitinib-induced thrombocytopenia, not observed with other antiangiogenic therapies. We discuss the differential diagnostic work-up, the necessary multidisciplinary approach, and the successful management of the case.

## 1. Introduction

Renal cell carcinoma (RCC) is the sixth most common cancer for men and eighth most common cancer for women in the USA [1]. Immunotherapies and antiangiogenic drugs are the main treatment options for patients who present with advanced and/or unresectable disease [2]. In most cases, metastatic RCC progresses despite first-line treatment, and usually a sequence of different anti‐vascular endothelial growth factor (VEGF) targeted therapies control the disease for a few years [3].

Axitinib is an oral and selective second-generation inhibitor of vascular endothelial growth factor receptor (VEGFR) 1, 2, and 3 [4]. It was approved in January 2012 for the treatment of metastatic RCC after failure of one systemic therapy and more recently in combination with pembrolizumab and avelumab for front-line treatment of metastatic RCC by the Food and Drug Administration [5–7].

Axitinib is in general well tolerated with approximately 30% of any adverse events of any grade, but with low frequency of significant myelosuppression including anemia (<1%), neutropenia (1%), thrombocytopenia (<1%), and lymphopenia (3%), when compared with other anti-VEGFR inhibitors (Tables 1 and 2) [5–14]. However, when they occur, they pose a significant challenge to treating physicians.

Herein, we present a patient with significant axitinib-induced thrombocytopenia; we discuss the differential diagnostic work-up and multidisciplinary management.

## 2. Case Presentation

In July 2008, a 70-year-old Caucasian man, who had Karnofsky performance score of 70%, with a significant medical history of grade 2 diastolic dysfunction, hyperlipidemia, hypertension, abdominal aortic aneurysm, coronary artery disease, esophagus stenosis, and hiatal hernia, all controlled with medication, presented with a 5 cm left renal mass of approximately invading the renal capsule, compatible with left RCC. The patient had no family history of cancer. The other drugs used by the patient were tadalafil, levothyroxine sodium, losartan potassium, amlodipine besylate, metoprolol, febuxostat, atorvastatin calcium, oxycodone HCl as needed, and acetylsalicylic acid. His social habits included sporadic alcohol consumption.

The patient underwent a left radical nephrectomy (08/2008); pathology revealed a pT1b clear cell renal cell carcinoma, nuclear grade II, without perirenal fat extension, and no adrenal gland involvement and negative margins (pT1b Nx Mx).

Approximately 6 years after his renal surgery (07/2014), the patient developed bilateral lower extremity weakness and walking difficulty, and diagnostic imaging revealed recurrent disease with thoracic spinal metastases (T6 and T7), causing cord compression at this level. He subsequently underwent corpectomy (T2–T9) and decompressive laminectomy (T6, T7, with a partial T5), and radiation therapy (XRT) was completed by August 2014. Pathology confirmed metastatic clear cell renal cell carcinoma. After radiation therapy, systemic treatment with pazopanib was initiated (09/2014).

The patient was on pazopanib 800 mg/day for two years with overall good tolerance including mild anemia and thrombocytopenia (grade I-II), but pazopanib dose was eventually decreased due to significant fatigue, diarrhea, and abdominal pain. Until June 2016, the patient was on pazopanib intermittently with GI toxicities and fatigue and eventually discontinued (06/2016) due to persistent toxicity.

He then received second-line nivolumab for a total of sixteen months but developed spinal progression at T5–T7 requiring decompressing surgery (04/2017) followed by XRT.

The patient was then successfully rechallenged with pazopanib from 06/2017 to 01/2019, with good tolerance on a lower dose of 600 mg/day and overall stable disease.

In January 2019, he again developed the progressive spinal disease at T5–T7 and underwent repeated surgical decompression. The patient was successfully treated with corticosteroids and was started on axitinib 5 mg BID.

After 10 days of starting axitinib, the patient developed severe fatigue (grade 4). He was observed in the outpatient clinic, and his complete blood count (CBC) revealed a platelet (Plt) count of 4 × 10^9^/liter. The baseline platelet count before treatment with axitinib was 245 × 10^9^/liter. Axitinib treatment was stopped, and the patient was admitted to the hospital for supportive therapy and work-up studies.

At this time, viral serology (hepatitis, CMV, and HIV) tests were tested negative. The patient's renal and hepatic function tests were in normal limits. Prothrombin time, INR and activated partial thromboplastin time, fibrinogen, and D-dimer levels were normal, and direct and indirect Coombs tests were negative. ADAMTS13 (a disintegrin and metalloprotease domain, with thrombospondin type 1 motif 13) activity test resulted 42.5% (normal greater than 10%), and the serotonin release assay was normal (<1). Disseminated intravascular coagulation (DIC), thrombotic thrombocytopenic purpura (TTP), and heparin-induced thrombocytopenia were excluded diagnoses.

**Figure 1:**
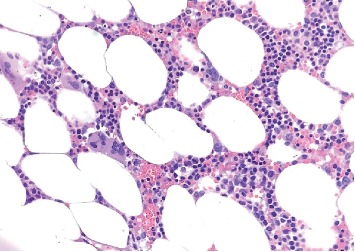
Biopsy shows normocellular bone marrow for age ∼30–40% with trilineage hematopoiesis without evidence of involvement by metastatic carcinoma or hematopoietic neoplasm. Megakaryocytes are normal in numbers and do not show significant atypia.

Abdominal ultrasound showed normal spleen dimensions. Bone marrow biopsy was performed which was normocellular, without metastatic disease, no increase blast (<1%), and no significant dysplasia. Megakaryocytes were in normal proportion (Figure 1).

The patient received supportive treatment with platelet transfusion (six units in total) with a good platelet transfusion response. Despite axitinib discontinuation, his platelet counts did not recover (12–15 × 10^9^/liter), and approximately one week later, he was started on a 4-day pulse of dexamethasone for possible immune thrombocytopenia, with an improvement of his platelet count to above 30 × 10^9^/liter after two days. His platelet counts continued to recover but stabilized at 88 × 10^9^/liter after 2 weeks of treatment. The second course of high-dose steroids was repeated to allow starting a new TKI for RCC.

The patient was eventually started on cabozantinib 40 mg with good tolerance and no worsening of thrombocytopenia. Interestingly, after two months of holding axitinib and while on cabozantinib, the platelet count increased to 136 × 10^9^/Liter. At the last visit, the patient was still on cabozantinib with clinical response and no other hematological events were observed.

## 3. Discussion

Herein, we describe a rare event of grade IV axitinib-induced thrombocytopenia, which was partially resolved with therapy discontinuation and high-dose steroids and not associated with exposure to other anti-VEGF inhibitors.

Thrombocytopenia in solid tumors can occur from bone marrow involvement with cancer, toxicity of anticancer treatments, or platelet consumption by DIC or TTP. Immune-related thrombocytopenia is rare in solid tumors [15]. Tumor cells can produce substances that induce platelet aggregation, and this aggregation causes immune-mediated destruction of platelets. Of note, there are few cases with RCC related ITP in literature. Furthermore, paraneoplastic syndrome can cause ITP in RCC patients regardless of stage [15]. The diagnosis of paraneoplastic ITP is one of the exclusion criteria supported by a normocellular bone marrow biopsy and platelet response after definitive treatment of the underlying RCC. In our case, thrombocytopenia occurred after starting a new drug and recovered after discontinuing the therapy and high-dose steroids, favoring the diagnosis of a drug-induced thrombocytopenia over a paraneoplastic syndrome.

In metastatic RCC, antiangiogenic TKIs are standard-of-care. VEGF-induced thrombocytopenia is rare reported in less than 10% with sunitinib and even less with newer generation TKIs [16]. There are several possible etiologies for TKI-induced thrombocytopenia, including the inhibition of KIT signaling, which is important for erythropoiesis, lymphopoiesis, and megakaryopoiesis; inhibition of VEGF, FLT-3, and PDGFR; and microangiopathy due to hypertension [16,17].

In a phase II trial in cytokine refractory RCC patients, there was no hematologic toxicity related with axitinib [18]. In another phase II trial, sorafenib refractory patients were also treated with axitinib. Grade I-II thrombocytopenia was reported in 19.6% of patients, and no grade ≥3 thrombocytopenia was reported in this study either [19]. Finally, in the AXIS trial, hematologic side effects were less frequent in axitinib arm when compared with sorafenib. Grade I-II thrombocytopenia occurred in 15% of patients, and only one patient had grade ≥3 thrombocytopenia [6]. Altogether in RCC studies with axitinib (*N* = 672), all grade thrombocytopenia occurred in about 1.6% of cases, grade 3 in 0.1%, and grade 4 in 0% [20].

Data reported in clinical trials are often not representative of the safety profile observed in real-world data. The exclusion criteria used in clinical studies of axitinib included ECOG ≤2 or Karnofsky performance score >70, grade III or IV heart failure, uncontrolled hypertension, active central nervous system metastasis, autoimmune disease, inadequate renal, hepatic, or hematologic organ function. Of note, our patient had none of these conditions. In addition, axitinib was not the first- or second-line treatment for this patient after prior-TKI. It is possible that prior angiogenic therapies may have caused thrombocytopenia, yet it did not occur with subsequent treatment with cabozantinib.

Drug-induced thrombocytopenia can be clinically significant, and significant thrombocytopenia has been previously reported with sunitinib in combination with trastuzumab in a patient with metastatic breast cancer after three weeks on treatment with both agents [17].

Other causes of thrombocytopenia have to be excluded for the final diagnosis of drug-induced thrombocytopenia. There is no test to confirm or rule out that the patient had ITP versus drug-induced thrombocytopenia. The treatment for drug-induced thrombocytopenia is to stop the culprit drug; however, steroids may be given to improve the total platelet count.

In this particular case, drug-induced thrombocytopenia is the most likely diagnosis because (1) there was a platelet count decrease (from normal to 4 × 10^9^/Liter) after 10 days of starting axitinib; (2) there was no interaction between axitinib and other drugs that are used by the patient [21] and (3) other causes such as infections, DIC, TTP, and bone marrow involvement were ruled out. In addition, after 2 months of discontinuation of axitinib and after a pulse of steroids, platelets increased to 136 × 10^9^/liter.

In conclusion, severe thrombocytopenia induced by axitinib is a rare but potentially life-threatening event and may be drug-specific rather than class-specific effect. A short-term pulse of corticosteroids and therapy discontinuation was associated with a clinical improvement and allowed further treatment with a different anti-VEGF therapy.

## Figures and Tables

**Table 1:** Hematologic adverse effects in major trials of Axitinib.

	Axi vs Placebo (Atlas) [8]	Axi vs. sorafenib (Front-line) [9]	Axi + pembrolizumab (KEYNOTE 426) [7]	Axi + avelumab (JAVELIN 101) [5]		Axi vs. sorafenib (AXIS) [6]	
	Any grade	Any grade	Any grade	Grade ≥3	All grades	Grade ≥3	Grade ≥3
Anemia	NR^*∗*^	NR	7.9	0.7	6	1.6	<1
Neutropenia	2	NR	1.9	0.2	1.4	0.2	1
Lymphopenia	NR^*∗*^	NR	NR	NR	NR	NR	3
Thrombocytopenia	2	NR	2.6	0	3.5	0.2	<1

NR: not reported; ^*∗*^<2%.

**Table 2:** Thrombocytopenia induced by VEGFR TKIs in RCC.

	Thrombocytopenia	
	All grades (%)	Grade III or IV (%)
Axitinib [6]	15	<1
Pazopanib [10]	41	3–1
Sunitinib [11]	65	8
Cabozantinib [12]	39.7	1.3
Sorafenib [6]	14	0
Tivozanib [13]	18	<1
Lenvatinib^*∗*^ [14]	NR	5

NR: not reported. ^*∗*^lenvatinib plus everolimus.
